# Putative Structural and Functional Coupling of the Mitochondrial BK_Ca_ Channel to the Respiratory Chain

**DOI:** 10.1371/journal.pone.0068125

**Published:** 2013-06-27

**Authors:** Piotr Bednarczyk, Mariusz R. Wieckowski, Malgorzata Broszkiewicz, Krzysztof Skowronek, Detlef Siemen, Adam Szewczyk

**Affiliations:** 1 Laboratory of Intracellular Ion Channels, Nencki Institute of Experimental Biology, Warsaw, Poland; 2 Department of Biophysics, Warsaw University of Life Sciences - SGGW, Warsaw, Poland; 3 Laboratory of Bioenergetics and Biomembranes, Nencki Institute of Experimental Biology, Warsaw, Poland; 4 Laboratory of Bioinformatics and Protein Engineering, International Institute of Molecular and Cell Biology, Warsaw, Poland; 5 Department of Neurology, Otto-von-Guericke-University, Magdeburg, Germany; 6 Laboratory of Molecular and Systemic Neuromorphology, Nencki Institute of Experimental Biology, Warsaw, Poland; University of Mississippi, United States of America

## Abstract

Potassium channels have been found in the inner mitochondrial membranes of various cells. These channels regulate the mitochondrial membrane potential, the matrix volume and respiration. The activation of these channels is cytoprotective. In our study, the single-channel activity of a large-conductance Ca^2+^-regulated potassium channel (mitoBK_Ca_ channel) was measured by patch-clamping mitoplasts isolated from the human astrocytoma (glioblastoma) U-87 MG cell line. A potassium-selective current was recorded with a mean conductance of 290 pS in symmetrical 150 mM KCl solution. The channel was activated by Ca^2+^ at micromolar concentrations and by the potassium channel opener NS1619. The channel was inhibited by paxilline and iberiotoxin, known inhibitors of BK_Ca_ channels. Western blot analysis, immuno-gold electron microscopy, high-resolution immunofluorescence assays and polymerase chain reaction demonstrated the presence of the BK_Ca_ channel β4 subunit in the inner mitochondrial membrane of the human astrocytoma cells. We showed that substrates of the respiratory chain, such as NADH, succinate, and glutamate/malate, decrease the activity of the channel at positive voltages. This effect was abolished by rotenone, antimycin and cyanide, inhibitors of the respiratory chain. The putative interaction of the β4 subunit of mitoBK_Ca_ with cytochrome c oxidase was demonstrated using blue native electrophoresis. Our findings indicate possible structural and functional coupling of the mitoBK_Ca_ channel with the mitochondrial respiratory chain in human astrocytoma U-87 MG cells.

## Introduction

Large-conductance Ca^2+^-regulated potassium channels (BK_Ca_ channels) are widely distributed in the plasma membranes of both excitable and non-excitable cells. BK_Ca_ channels are activated by membrane depolarization and the elevation of the intracellular calcium ion concentration. The basic component of a functional BK_Ca_ channel is the α subunit, which is encoded by a single gene (KCNMA1 or *Slo1*). Four monomers of the α subunit create the pore of the channel. Modulatory β1-β4 subunits (encoded by KCNMB1, KCNMB2, KCNMB3 or KCNMB4) can be associated with this tetramer.

A similar potassium channel was discovered in the inner mitochondrial membrane. The mitochondrial large-conductance Ca^2+^-regulated potassium channel (mitoBK_Ca_ channel) was first described in the human glioma cell line LN229 [1]. This channel can be blocked by charybdotoxin (ChTx) and stimulated by micromolar Ca^2+^ concentrations. In addition, this channel is regulated by the membrane potential. The mitoBK_Ca_ channel has also been identified in mitochondria of guinea pig ventricular cells [2]. This channel, which has properties similar to those of the plasma membrane BK_Ca_ channel, can be stimulated by the potassium channel opener NS1619 and can be blocked by iberiotoxin (IbTx) and paxilline. Additionally, it has been reported that the mitoBK_Ca_ channel is modulated by cAMP-dependent kinase, which depolarizes the membrane potential and attenuates the mitochondrial calcium overload [3]. To determine the subcellular localization and distribution of this channel, rat brain fractions were examined by Western blotting, immunocytochemistry and immuno-gold electron microscopy. These studies provided concrete morphological evidence for the existence mitoBK_Ca_ channel subunits in brain mitochondria fractions [4], [5].

Other potassium channels, including ATP-regulated (mitoK_ATP_ channel), intermediate-conductance Ca^2+^-regulated (mitoIK_Ca_ channel), voltage-gated (mitoKv channel) and pH-sensitive rectifying channels, are present in the inner mitochondrial membranes of different cell types [6], [7], [8], [9]. These channels affect mitochondrial matrix swelling, regulate the concentrations of reactive oxygen species (ROS) and change the mitochondrial membrane potential. It should be noted that mitochondrial K^+^ flux and subsequent ROS generation occur without changes in the membrane potential [10]. Additionally, it has been suggested that mitochondrial potassium channels are triggers of cytoprotection, but the mechanisms of this process are still under investigation [7], [11], [12], [13].

A recent study demonstrated that mitoK_ATP_ (ROMK) channels confer protection against cell death [14]. The identification of mitoROMK channels provided for the first time a molecular target for mechanistic and therapeutic investigations of this cell survival pathway. These data confirm the general hypothesis that mitochondrial potassium channels, such as mitoK_ATP_ or mitoBK_Ca_ channels, are cytoprotective [14]. Whether the mitoK_ATP_ channel plays a preferential role in ischemic preconditioning is still an open question [15].

The goal of this study was to identify the possible structural and functional coupling of the respiratory chain to the mitoBK_Ca_ channel in human astrocytoma mitochondria. For this purpose, we employed the patch-clamp technique, RT-PCR, Western blotting, immunocytochemistry techniques, immuno-gold staining and high-resolution blue native and SDS-PAGE 2D separation of mitochondrial proteins. Our findings confirm that mitochondrial BK_Ca_ channels with properties similar to those of the surface membrane BK_Ca_ channel are present in human astrocytoma mitochondria and can be regulated by the redox status of the respiratory chain via coupling to cytochrome c oxidase (complex IV).

## Materials and Methods

### Astrocytoma U-87 MG Cell Line and Isolation of Mitochondria

Cells were cultured in DMEM supplemented with 10% FCS, 2 mM L-glutamine, 100 U/ml penicillin, and 100 µg/ml streptomycin at 37°C in a humidified atmosphere with 5% CO_2_. The cells were fed and reseeded every third day.

The cells’ identity was confirmed using the short tandem repeat (STR) profiling technique. This assay was performed according to recently published guidelines [16], [17]. Brieﬂy, cells were expanded and frozen at 90% conﬂuence during the exponential growth phase and sent for STR profiling analyses to the German Collection of Microorganisms and Cell Cultures (Leibniz Institut Deutsche Sammlung für Mikroorganismen und Zellkulturen (DSMZ), Braunschweig, Germany).

Mitochondria were prepared from the human astrocytoma (glioblastoma) U-87 MG cell line as previously described [18]. Human astrocytoma cells from five culture flasks were collected in PBS medium and centrifuged at 800 g for 10 min. The cell pellet was resuspended and homogenized in a preparation solution (250 mM sucrose, 5 mM HEPES, pH = 7.2). To isolate the mitochondria, the homogenate was centrifuged at 9 200 g (10 min). The pellet was then suspended and centrifuged at 780 g (10 min). The supernatant was transferred to a new tube and centrifuged at 9 200 g for 10 min. The pelleted mitochondria were then resuspended in storage solution (150 mM KCl, 10 mM HEPES, pH = 7.2) and centrifuged at 9 200 g for 10 min. Finally, the mitochondria were resuspended in 0.3 ml of storage solution. All procedures were performed at 4°C. The solution used for the isolation of mitochondria for Western blot analysis and blue native PAGE was further supplemented with 1% BSA and a protease inhibitor cocktail (Roche).

### Patch-clamp Experiments

**Figure 1:**
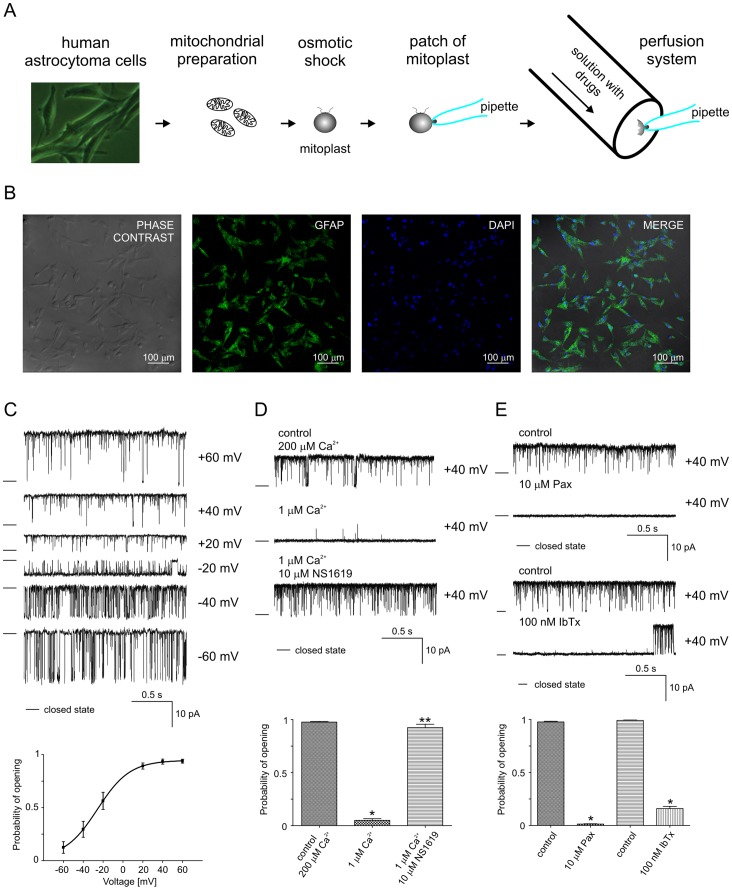
Identification of the mitoBK_Ca_ channel in human astrocytoma mitochondria using the patch-clamp technique. **A.** Schematic representation of the mitoplast preparation, patching of the mitoplast, and, finally, the patch-clamp experiment in the inside-out mode by means of the perfusion system. The matrix side of mitochondrial membrane is exposed to externally added substances. For details of patch-clamp measurements see the *Materials and Methods* section. The image on the left shows cultured human astrocytoma U-87 MG cells (phase contrast). **B.** High-power confocal image of immunolabeling for GFAP (green) in cultured human astrocytoma cells. The DNA-binding dye DAPI was used to stain the cell nuclei (blue). Superimposition of the GFAP and DAPI signals and the phase contrast image of the cells confirmed the purity of the cell culture used in our experiments. **C.** Single-channel recordings in symmetric 150/150 mM KCl isotonic solution (200 µM Ca^2+^) at different voltages. The P_o_ of the mitoBK_Ca_ channel under control conditions at different voltages (solid line, ▪). **D.** Single-channel recordings in symmetric 150/150 mM KCl isotonic solution show the influence of Ca^2+^ and NS1619 on channel activity. The current traces at 200 µM Ca^2+^ (control, upper trace) and at 1 µM Ca^2+^ (middle trace) demonstrate the decrease in the single-channel activity at decreasing Ca^2+^ concentrations. This effect is reversible upon the addition of 10 µM NS1619 (lower trace). The panel below shows P_o_ under the conditions above (n = 3). *P<0.001 vs. the control. **P<0.001 vs. 1 µM Ca^2+^. **E.** Effects of 10 µM paxilline (Pax) and 100 nM iberiotoxin (IbTx) on the single-channel activity. The distribution of the probability of channel opening under the above conditions is shown below the graph (n = 3). *P<0.001 vs. the control.

Patch-clamp experiments using mitoplasts were performed as described previously [18], [19]. Briefly, mitoplasts were prepared from a sample of human astrocytoma mitochondria placed in a hypotonic solution (5 mM HEPES, 200 µM CaCl_2_, pH = 7.2) for approximately 1 min to induce swelling and breakage of the outer membrane. Then, a hypertonic solution (750 mM KCl, 30 mM HEPES, 200 µM CaCl_2_, pH = 7.2) was added to restore the isotonicity of the medium. The patch-clamp pipette was filled with an isotonic solution containing 150 mM KCl, 10 mM HEPES, and 200 µM CaCl_2_ at pH = 7.2. Mitoplasts are easily recognizable due to their size, round shape, transparency, and presence of a “cap”, characteristics that distinguish these structures from the cellular debris that is also present in the preparation. An isotonic solution containing 200 µM CaCl_2_ was used as the control solution for all of the presented data. The low-calcium solution (1 µM CaCl_2_) contained the following: 150 mM KCl, 10 mM HEPES, 1 mM EGTA and 0.752 mM CaCl_2_ at pH = 7.2. All of the modulators of the channels and the substrates and inhibitors of the respiratory chain were added as dilutions in isotonic solution containing 200 µM CaCl_2_. To apply these substances, we used a perfusion system containing a holder with a glass tube (made in our workshop), a peristaltic pump, and Teflon tubing. The mitoplasts at the tip of the measuring pipette were transferred into the openings of a multibarrel “sewer pipe” system in which their outer faces were rinsed with the test solutions (Fig. 1A). The configuration of our patch-clamp mode is presented in Fig. 1A. The experiments were carried out in patch-clamp inside-out mode. This is based on observations with various mitochondrial substrates applied such as NADH or succinate. Reported voltages are those applied to the patch-clamp pipette interior. Hence, positive potentials represent the physiological polarization of the inner mitochondrial membrane (outside positive).

The electrical connection was made using Ag/AgCl electrodes and an agar salt bridge (3 M KCl) as the ground electrode. The current was recorded using a patch-clamp amplifier (Axopatch 200B, Molecular Devices Corporation, USA). The pipettes, made of borosilicate glass, had a resistance of 10–20 MΩ and were pulled using a Flaming/Brown puller.

The currents were low-pass filtered at 1 kHz and sampled at a frequency of 100 kHz. The traces of the experiments were recorded in single-channel mode. The illustrated channel recordings are representative of the most frequently observed conductance for the given condition. The conductance of the channel was calculated from the current-voltage relationship (data not shown). The probability of channel opening (P_o_, open probability) was determined using the single-channel search mode of the Clampfit 10.2 software. Calculations were performed using segments of continuous recordings lasting 60 s, with N>1000 events. Data from the experiments are reported as the mean values ± standard deviations (S.D.). Student’s t-test was used for statistical analysis. In figures showing single-channel recordings, “-” indicates the closed state of the channel.

### Immunostaining for Glial Fibrillary Acidic Protein (GFAP)

The cells were fixed in 4% PFA at room temperature (30 min), rinsed in PBS and incubated with 50 mM NH_4_Cl in PBS (15 min). After washout, the cells were subjected to blocking/permeabilisation solution (DSB) containing 5% NDS, 0.075% saponin and 1% BSA diluted in PBS (30 min). Then, the cells were incubated with DSB solution containing an anti-GFAP antibody (anti-GFAP, 1∶200, Abcam). The immunoreaction was visualized using the secondary antibody Alexa-Fluor 488 (1∶200, Molecular Probes). To visualize the cell nuclei, the DNA binding dye DAPI (Vector Laboratories) was used. Confocal images were acquired using a model TCS SP5 Leica microscope (Leica Microsystem, Wetzlar, Germany).

### cDNA Synthesis and Polymerase Chain Reaction

The total RNA from human astrocytoma U-87 MG cells was prepared using an RNeasy Mini Kit (Qiagen) (n = 7). RNA was reverse transcribed using the RevertAid™ First Stand cDNA Synthesis Kit (Fermentas). The reverse transcription mixture with 3 µg total RNA (∼5 µl – depend on preparation RNA) was mixed with 1 µl oligo(dT)_18_ primer and filled DEPC-treated water up to 12 µl, then was added 4 µl 5× Reaction Buffer, 1 µl RiboLock™ RNase Inhibitor, 2 µl dNTP Mix (10 mM) and 1 µl RevertAid™ M-MuLV Reverse Transcriptase (200 u/µl). Total volume was equal to 20 µl. The reactions were run for 60 min at 42°C and terminated by incubation for 5 min at 70°C. Parallel cDNA synthesis reactions were run in the absence of the RevertAid™ M-MuLV Reverse Transcriptase to assess the level of genomic DNA contamination in the RNA sample.

The resulting cDNA was used as a DNA template for PCR amplification. PCR was conducted with the following primers: BK_Ca_ β1 (accession No. NM_019273), forward primer 5′GTGACTCCATGCTGCTGTG 3′, reverse primer 5′ CACACAGAAGACACTCGGGA 3′; BK_Ca_ β2 (accession No. NM_176861), forward primer 5′ CTGGGAATCACACTGCTGC 3′, reverse primer 5′ GAAGTGTTGGTGTCTCCTGAAG 3′; BK_Ca_ β3 (accession No. NM_001104560), forward primer 5′ CTGTAGAACCACCCAAGTCC 3′, reverse primer 5′ ACCCTGTAGTGTGATTTGGAC 3′; BK_Ca_ β4 (accession No. NM_023960), forward primer 5′CGAAGACAAGAGCATCCG3’, reverse primer 5′ CAAGTGAATGGCTGGGAAC 3′; and GAPDH (accession No. NM_002046.3), forward primer 5′ CAAGGTCATCCATGACAACTTTG 3′, reverse primer 5′ GTCCACCACCCTGTTGCTGTAG 3′. The sizes of the PCR product were as follows: BK_Ca_ β1, 336 bp; BK_Ca_ β2, 324 bp; BK_Ca_ β3, 300 bp; BK_Ca_ β4, 405 bp; and GAPDH, 496 bp. The PCR amplification was performed as follows: initial denaturation at 95°C for 3 min; denaturation at 95°C for 30 s; primer annealing at 59.5°C (for the BK_Ca_ β1, β3, and β4 primers) or 62.5°C (for BK_Ca_ β2 primers) for 3 minutes; extension at 72°C for 1 min; 35 cycles at 95°C for 30 s, at 54°C for 30 s, and at 72°C for 1 min; and a final extension step at 72°C for 15 min (Thermal Cycler C1000™, BioRad). The PCR products were separated by electrophoresis on a 1% agarose gel and were visualized with ethidium bromide.

### SDS-PAGE and Western Blot Analysis

Homogenates of human astrocytoma U-87 MG cells and brain tissue and mitochondria fractions were solubilized with RIPA buffer (50 mM Tris, 150 mM NaCl, 0.1% SDS, 0.5% Na deoxycholate, 1% Nonidet P-40, 1 mM EGTA, 1 mM EDTA, 1 tab. Complete Mini/10 ml). Samples containing 30 µg of protein were separated by 12% sodium dodecyl sulfate-polyacrylamide gel electrophoresis (SDS-PAGE) and transferred onto 0.45 µm nitrocellulose membranes (BioRad). The membranes were exposed to polyclonal antibodies that recognize BK_Ca_ channel β subunit 4 (anti-β4, 1∶200, Alomone Labs) and cytochrome c oxidase subunit IV (COX IV, 1∶1000, Cell Signaling). The specificity of the BK_Ca_ β4 subunit antibody was confirmed by specific blocking peptide experiments in which a mixture of the primary antibody and an immunogenic peptide was applied. The blocking peptide for the anti-β4 subunit antibody was provided as a control antigen by Alomone Labs. The blots were developed using a secondary anti-rabbit antibody coupled to horseradish peroxidase in conjunction with ECL (GE Healthcare UK Limited).

### Immuno-gold Electron Microscopy

The post-embedding procedure was performed essentially as described by Mathiisen et al. [20]. Human astrocytoma cells were fixed with 4% paraformaldehyde plus 0.1% glutaraldehyde. The cells were pelleted, cryoprotected in 30% sucrose in 0.01 M PBS, and frozen using an EM PACT high-pressure freezer (Leica). To optimally preserve both the antigenicity and the ultrastructure, the frozen samples were subjected to osmium-free freeze substitution followed by Lowicryl HM20 (Polysciences, Inc.) embedding and UV-induced polymerization at −50°C using an EM ASF2 apparatus (Leica). Ultrathin sections were cut using an ultramicrotome (Leica Ultracut R; Leica) and mounted on mesh nickel grids coated with Formvar 300 (Agar Sciences). The immunoreactions consisted of sequential incubations with a rabbit polyclonal anti-BK_Ca_ channel β subunit 4 antibody (anti-β4, 1∶20, Alomone Labs) followed by species-specific donkey secondary antibodies coupled to 10 nm gold particles (Electron Microscopy Sciences). The sections were counterstained with uranyl acetate. In control experiments, the primary antibody was replaced with an irrelevant rabbit immunoglobulin. The specimens were examined using an electron microscope (JEM 1400 high-resolution transmission electron microscope, JEOL Co.) at 120 kV. No labeling with gold particles was observed for samples in which the primary antibody (anti-β4, 1∶20, Alomone Labs) was not used (data not shown).

### Construction of the pKCNB4_GFP Plasmid

The KCNMB4 coding sequence (accession No. NM_023960) was synthesized commercially by GenScript with a SalI site preceding the initiation codon and an EcoRI site after the codon for serine 210, the last amino acid in the native protein. The synthesized gene was cleaved with SalI and EcoRI and cloned into pEGFP-N1 (Clontech), resulting in the plasmid pKCNMB4_GFP, which expresses the KCNMB4 protein with GFP fused to the C-terminus. The success of these manipulations was confirmed by DNA sequencing.

### Transfection Assays and Fluorescence Staining of Cultured Astrocytoma Cells

The β4-GFP expression plasmid pKCNMB4_GFP was used in transfection assays with human astrocytoma cells. The cells were transfected at 60–70% confluence. Standard transfection assays were performed using Lipofectamine (Invitrogen) as described by the manufacturer. In brief, Lipofectamine-DNA complexes were formed by mixing 4 µl of Lipofectamine with 1 µl (200 ng) of the plasmid pKCNMB4_GFP. The cells were incubated for 3 h in transfection medium. Then, the medium was replaced with fresh medium, and the cells were further cultured. After 12 h, the cells were fixed in 3% PFA at room temperature (30 min), rinsed in PBS and incubated with 50 mM NH_4_Cl in PBS (15 min). After being washed, the cells were incubated in blocking/permeabilisation solution (DSB) containing 5% NDS, 0.075% saponin and 1% BSA diluted in PBS (30 min). Then, the cells were incubated in DSB solution containing an anti-OxPhos complex IV subunit I primary antibody (OxPhos, 1∶200, Invitrogen). The immunoreaction was visualized using an Alexa-Fluor 555-conjugated donkey anti-mouse antibody (1∶200, Molecular Probes). To visualize the cell nuclei, the DNA binding dye DAPI (Vector Laboratories) was used. Confocal images were acquired using a model TCS SP5 Leica microscope (Leica Microsystem, Wetzlar, Germany).

### Blue Native Electrophoresis (BNE) and SDS-PAGE 2D Separation of Mitochondrial Proteins

Mitochondria isolated from human astrocytoma cells were solubilized in 1.5 M aminocaproic acid, 50 mM Bis-Tris-HCl, pH = 7.0 and 1% dodecylmaltoside. The samples were incubated on ice for 20–30 min and then centrifuged at 10 000 g for 15 min to remove the unsolubilized material. The protein concentrations in the supernatants were determined using the Bradford method with a protein assay kit (Bio Rad). Supernatant samples containing 60 µg of mitochondrial protein were combined with 1 µl of 5% Coomassie brilliant blue (in 1.5 M aminocaproic acid, 50 mM Bis-Tris-HCl, pH = 7.0) and separated on a large-format (1 mm/16 cm/20 cm) 5–12% gradient acrylamide gel in the first dimension. To assess the quality of the sample separation in the first-dimension electrophoresis step and to calibrate the BNE gel (in kDa), samples containing 30 µg of rat heart mitochondria were processed as described for mitochondria isolated from human astrocytoma cells. After the first electrophoresis step, the 1st-dimension BNE gel lines (corresponding to the separated individual samples) were equilibrated in a solution containing 2% SDS and 5 mM tributylphosphine (for the reduction of cysteines) for 15 min and then in a solution containing 2% SDS and 260 mM iodoacetamide (for the alkylation of cysteines) for another 15 min. Next, the 1st-dimension BNE gel lines were stacked over a 10% SDS-PA gel, separated and transferred to a PVDF membrane. The membranes were immunoblotted for β4 (anti-β4, 1∶200, Alomone Labs) and then for respiratory chain subunits (OXPHOS, 1∶5 000, MitoSciences). After hybridization with a peroxidase (HRP)-conjugated secondary antibody, the signal was revealed using the ECL Plus Western blot detection reagent (Amersham Pharmacia Biotech).

## Results

The experiments described in this report were performed in three different sets:

- Functional and molecular characterization of the BK_Ca_ channels present in human astrocytoma mitochondria.
- Functional coupling of mitoBK_Ca_ channels to the respiratory chain.
- Putative structural coupling of mitoBK_Ca_ channels to cytochrome c oxidase.

### A1. Identification of Mitochondrial BK_Ca_ Channels in Astrocytoma Cells using Patch-clamping

Patch-clamp experiments were performed with mitoplasts from human astrocytoma U-87 MG cells (Fig. 1A). The cells’ identities were confirmed as described in the *Materials and Methods*. Immunocytochemical labeling of glial fibrillary acidic protein (GFAP) with anti-GFAP antibodies revealed full localization of GFAP in our cultured cells. Additionally, DAPI staining and phase contrast imaging were performed (Fig. 1B). The current was measured in a symmetric 150/150 mM KCl isotonic solution with a CaCl_2_ concentration of 200 µM. In n = 120 patches, we observed a channel with a single-channel conductance of 290±10 pS as calculated based on the mean of the current-voltage relationship. Fig. 1C shows the single-channel recordings and the probabilities of channel opening at different voltages. No rectification of the current was observed (data not shown). The open probability (P_o_) increased from ∼0.15 at −60 mV to ∼0.95 at positive voltages (Fig. 1C, lower panel). To test the Ca^2+^ sensitivity of the channel, we reduced the Ca^2+^ concentration from high (200 µM) to low (1 µM). Figure 1D (upper panel) displays single-channel recordings at a holding potential of +40 mV and Ca^2+^ concentrations of 200 µM (control) and 1 µM. These recordings demonstrate an immediate strong inactivating effect of the low Ca^2+^ concentration. The analysis of P_o_ revealed that the inhibition was statistically significant. After the addition of the BK_Ca_ channel opener NS1619, P_o_ increased from 0.05 to 0.95 (Fig. 1D, lower panel).

Substances known to be inhibitors of mitoBK_Ca_ channel activity were also tested. Figure 1E (upper and middle parts) illustrates the activity of the channel under the control conditions after the application of 10 µM paxilline (Pax) and 100 nM iberiotoxin (IbTx) in separate experiments. Paxilline and IbTx inhibited the channel activity. P_o_ decreased from 0.95 to 0.01 for Pax and from 0.95 to 0.15 for IbTx after the application of the drug (Fig. 1E, lower panel). Our observations suggest that both, calcium and IbTx binding site are probably present at the matrix side of the inner mitochondrial membrane. A detailed topology of the mitoBK_Ca_ channel has to be established in further studies. Typically, the experiments for detecting mitoBK_Ca_ channels and testing drugs were finished after the application of 10 µM paxilline. Inhibition was always observed. Taken together, the measured single-channel conductance, the voltage- and Ca^2+^-dependent regulation, and the sensitivity to selective agonists and antagonists strongly indicate that our recordings correspond to a Ca^2+^-regulated potassium channel of the BK type, thus indicating the presence of this type of channel in astrocytoma cell mitochondria.

### A2. Detection of BK_Ca_ Channel Regulatory β4 Subunit mRNA in Astrocytoma Cells

**Figure 2:**
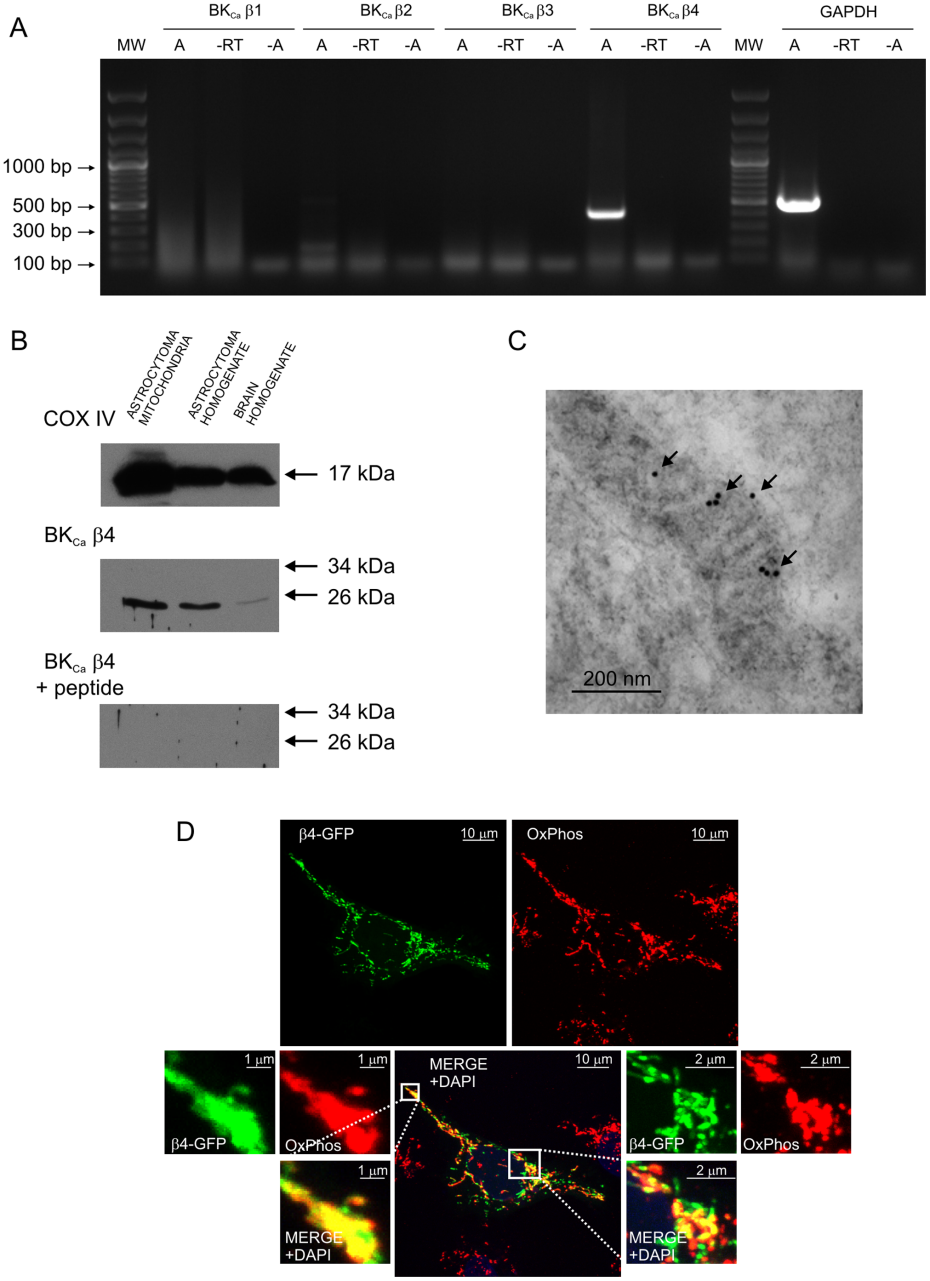
Localization of the BK_Ca_ channel regulatory β4 subunit in astrocytoma mitochondria. **A.** Detection of mitoBK_Ca_ channel regulatory β4 subunit mRNA in astrocytoma cells. The BK_Ca_ subunit β4 mRNA was detected at a size of 405 bp. No products were obtained for the BK_Ca_ subunits β1, β2 and β3. GAPDH served as a positive control and was detected at a size of 496 bp. The negative control without reverse transcriptase (−RT) and samples without cDNA (−A) had no signals. The results presented are representative of seven independent experiments. **B.** Immunoblot of astrocytoma mitochondria, astrocytoma cell homogenate and brain homogenate fractions labeled with the anti-BK_Ca_ channel β4 subunit antibody. A control antigen (BK_Ca_ β4+ peptide) was used as a positive control for the specificity of the antibody. An anti-cytochrome c oxidase subunit IV antibody (COX IV) was used as a mitochondrial marker (n = 3). **C.** Immuno-gold electron microscopy localization of the BK_Ca_ channel β4 regulatory subunit in mitochondria of cultured human astrocytoma cells. The β4 subunit molecules were labeled using 10 nm colloidal-gold particles (arrows). **D.** High-power confocal image of cultured astrocytoma cells immunolabeled to detect OxPhos (red) and β4-GFP-transfected cells (green). The superimposition of the two signals revealed the mitochondrial localization of BK_Ca_ β4 in human astrocytoma cells (yellow). The DNA-binding dye DAPI was used to stain the cell nuclei (blue). For details concerning the astrocytoma cells, see the *Materials and Methods*.

The expression of all four BK_Ca_ channel regulatory β subunits in human astrocytoma cells was investigated using a reverse transcription reaction technique (Fig. 2A). A strong signal for the BK_Ca_ channel β4 product (but not β1-β3 the products) was detected at a size of 405 bp. In parallel, GAPDH, the positive control, was detected at a size of 496 bp. To confirm the purity of the RNA preparation used, we performed negative controls using samples without reverse transcriptase (−RT) and samples without cDNA (−A). In both cases, the signal for the BK_Ca_ channel β subunit was not detected. Our findings indicate that the BK_Ca_ channel β4 subunit may be expressed in astrocytoma cells.

### A3. Immunodetection of the BK_Ca_ Channel Regulatory β4 Subunit in Astrocytoma Mitochondria

To investigate the expression of the mitochondrial BK_Ca_ channel regulatory subunit, we employed the Western blot (WB) technique, and we analyzed the presence of the BK_Ca_ channel β4 subunit in mitochondria from the human astrocytoma cell line. To determine the purity of the mitochondrial fraction, we used antibodies against cytochrome c oxidase subunit IV (COX IV), a marker of the inner mitochondrial membrane. As is shown in Figure 2B, we detected an increase of the COX IV signal immunoreactivity in the mitochondrial fraction versus the total astrocytoma cell homogenate sample. This result confirmed the quality and the enrichment of the mitochondria, indicating that this fraction was suitable for further immunodetection studies. As demonstrated by WB analysis, the BK_Ca_ channel β4 regulatory subunit was detected in the astrocytoma mitochondria. Similar to the results of Piwonska et al. [5], a specific band closely below 26 kDa was detected (Fig. 2B). To confirm that the observed labeling was specific, an additional WB was performed using a peptide that blocked the specific interaction between the antibody and the antigen, and no labeling was detected. In addition, to confirm the reactivity of the anti-β4 antibody in human astrocytoma mitochondria, brain tissue known to express the β4 regulatory subunit was used as a control. This sample showed an immunoreactive product, and a bright band was observed at the same level, just below 26 kDa (Fig. 2B).

### A4. Immuno-gold Staining of the β4 Subunit of mitoBK_Ca_ in Astrocytoma Cells

To confirm the results obtained using Western blot analysis, immuno-gold electron microscopy was performed on human astrocytoma cultures. The 10 nm gold particles were detected in mitochondria, confirming the presence of the BK_Ca_ channel β4 subunit in the mitochondrial membranes (Fig. 2C). Labeling with gold particles was observed in other cellular compartments (data not shown).

### A5. Immunofluorescence Analysis of the Distribution of the β4 Subunit of mitoBK_Ca_ in Astrocytoma Cells

To determine the subcellular localization of the BK_Ca_ channel β4 subunit in astrocytoma cells and to assess the association of this subunit with mitochondria, we determined the distribution of the β4 subunit using a β4-GFP protein and stained mitochondria using an antibody against cytochrome c oxidase. Transfections were performed using Lipofectamine to deliver the pKCNMB4_GFP plasmid to the human astrocytoma cells. The β4-GFP signal and the immunocytochemical labeling performed with antibodies against anti-OxPhos complex IV subunit I (OxPhos, a mitochondrial marker) revealed partial localization of the β4 subunit in the mitochondria (Fig. 2D). At a higher magnification, it was apparent that part of the β4-GFP signal overlapped with the mitochondrial staining. These observations indicate that the BK_Ca_ regulatory β4 subunit is localized to the astrocytoma mitochondria.

To assess the functional coupling of the respiratory chain with the mitoBK_Ca_ channel, a set of experiments using mitochondrial substrates (and inhibitors of mitochondrial respiration) were performed. Following the activity of the mitoBK_Ca_ channel using the patch-clamp method in this type of experiment provides a unique methodological opportunity. These experiments are based on clamping the membrane potential of the mitochondria (in the absence and presence of mitochondrial substrates) to a fixed value.

### B1. Regulation of the mitoBK_Ca_ Channel by NADH

**Figure 3:**
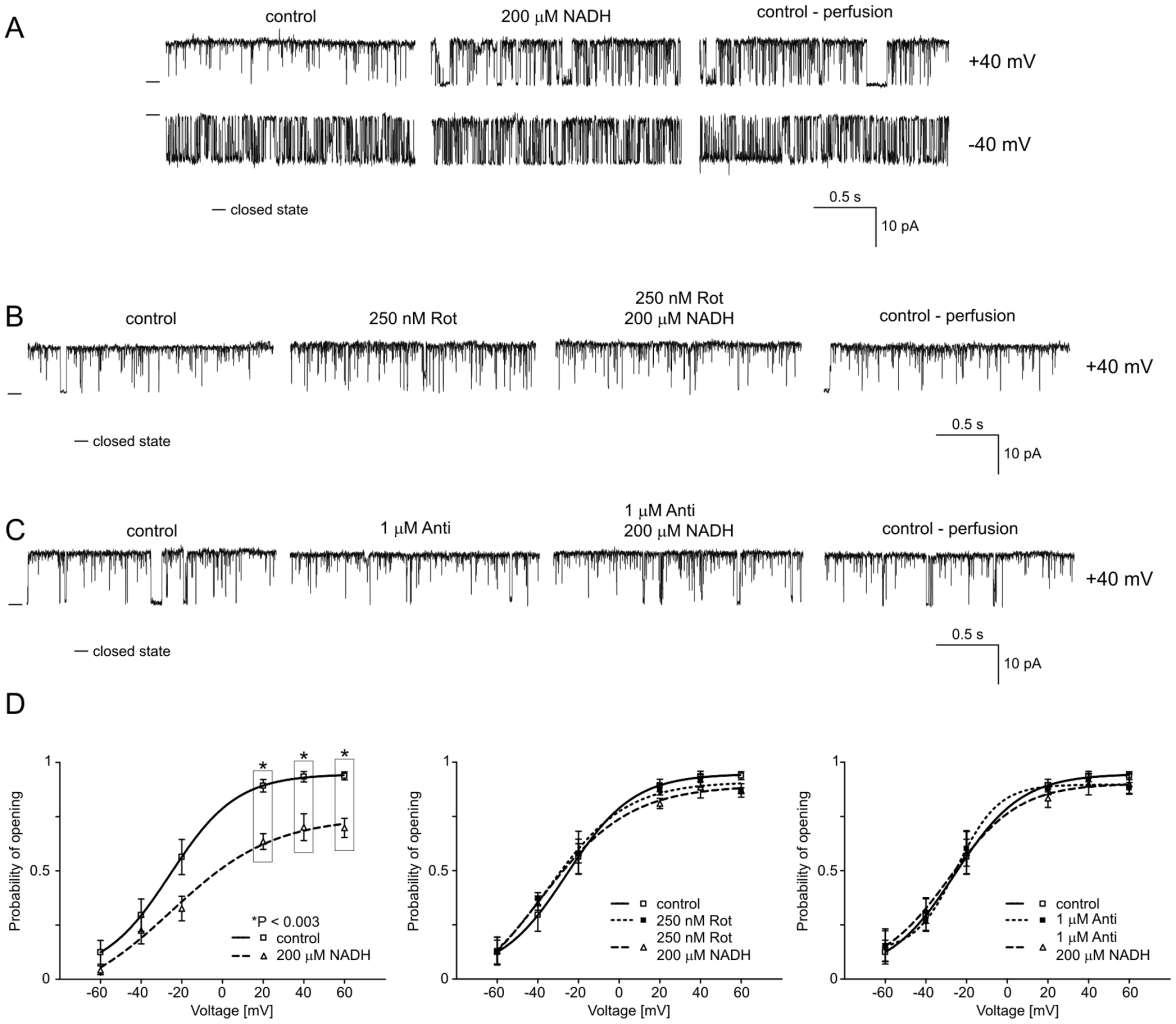
NADH reduces the P_o_ of the mitochondrial large-conductance Ca^2+^-regulated potassium channel at positive voltages. **A.** Single-channel recordings of the mitoBK_Ca_ channel activity in symmetric 150/150 mM KCl isotonic solution (200 µM Ca^2+^) at +40 and −40 mV under control conditions, after the addition of 200 µM reduced nicotinamide adenine dinucleotide (NADH) and after perfusion. **B.** Single-channel recordings of the mitoBK_Ca_ channel activity in a symmetric 150/150 mM KCl isotonic solution (200 µM Ca^2+^) at +40 mV under control conditions, after the addition of 250 nM rotenone (Rot) and 200 µM NADH plus 250 nM Rot, and after perfusion. **C.** Single-channel recordings of the mitoBK_Ca_ channel activity in symmetric 150/150 mM KCl isotonic solution (200 µM Ca^2+^) at +40 mV under control conditions, after the addition of 1 µM antimycin A (Anti) and 200 µM NADH plus 1 µM Anti and after perfusion. **D.** Analysis of P_o_ under the conditions described in A, B and C. *P<0.003 vs. the control.

A set of experiments confirmed the presence of BK_Ca_ channels in mitochondria of astrocytoma cells (see A1). As the first substrate of respiratory chain complex I, we used the reduced form of nicotinamide adenine dinucleotide (NADH). Figure 3A shows selected current-time traces of the mitoBK_Ca_ channel activity in symmetrical isotonic solution at different voltages after the addition of 200 µM NADH and during perfusion with control solution. This figure shows that NADH reduced P_o_ at positive voltages, and the effect was irreversible (n = 4). The analysis of P_o_ indicated that the changes were statistically significant at positive voltages (Fig. 3D, left-hand panel). To determine if the NADH-mediated inhibition of the mitoBK_Ca_ channel is due to a direct interaction between this substance and the mitochondrial channel or if this inhibition involves other proteins in the respiratory chain, we used inhibitors of respiratory chain enzymes. Rotenone (Rot, 250 nM), an inhibitor of NADH dehydrogenase (complex I), or antimycin A (Anti, 1 µM), an inhibitor of ubiquinol cytochrome c oxidoreductase (complex III), was applied before the addition of NADH. Figure 3B and C present selected current-time traces of mitoBK_Ca_ channel activity at +40 mV (n = 3 for each experiment). The effects of NADH in the presence of these inhibitors of the respiratory chain were evaluated. Additionally, the distribution of P_o_ is shown (Fig. 3D, middle and right panels). This result suggests that NADH regulates the mitoBK_Ca_ channel activity via an interaction with other complexes in the respiratory chain and not via a direct interaction (as a ligand) with the mitochondrial channel. Hence, subsequent experiments were performed with other substrates of the mitochondrial respiratory chain.

### B2. Regulation of the mitoBK_Ca_ Channel by Glutamate/malate

**Figure 4:**
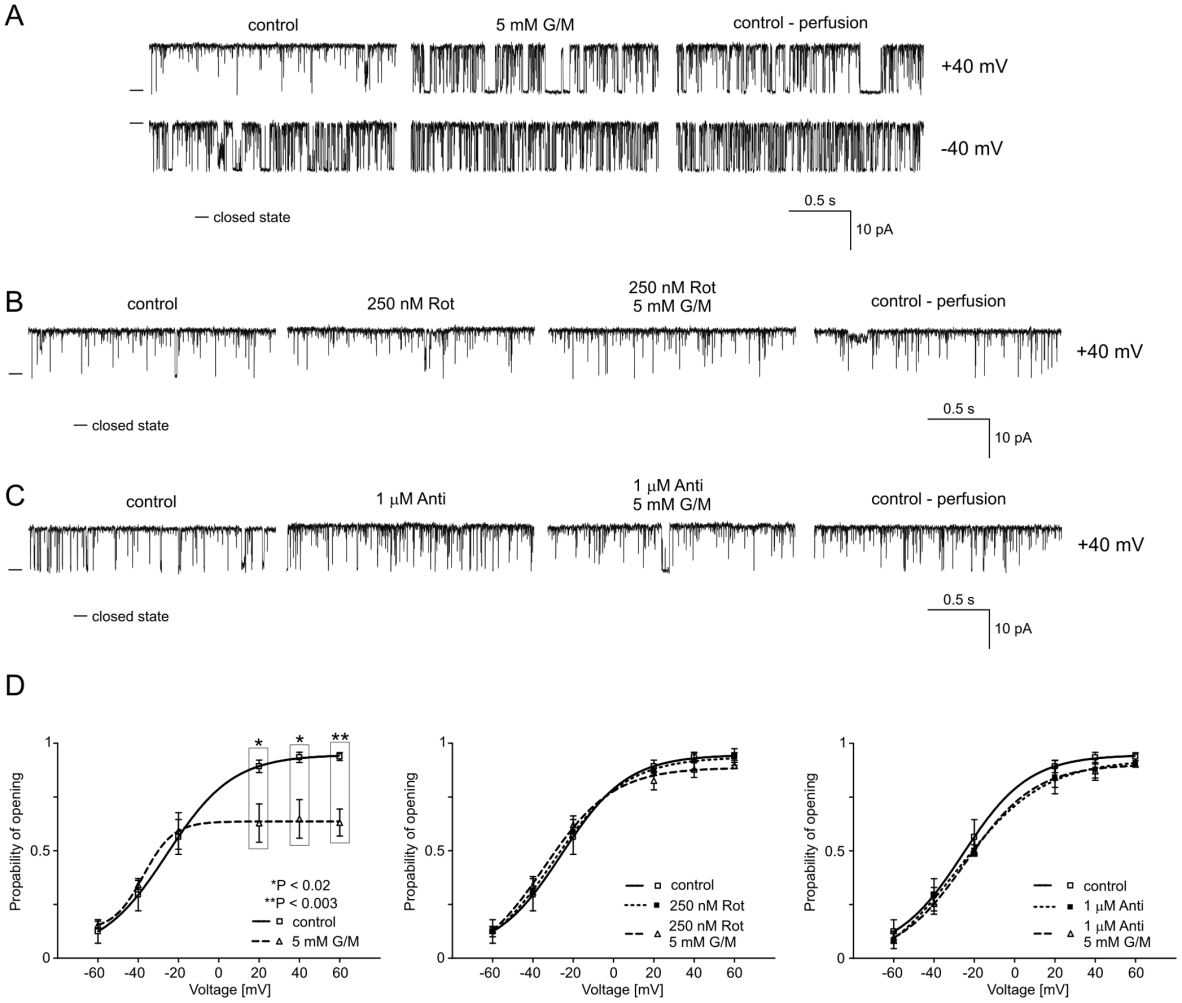
Glutamate/malate reduces the P_o_ of the mitochondrial large-conductance Ca^2+^-regulated potassium channel at positive voltages. **A.** Single-channel recordings of the mitoBK_Ca_ channel activity in symmetric 150/150 mM KCl isotonic solution (200 µM Ca^2+^) at +40 and −40 mV under control conditions, after the addition of 5 mM glutamate/malate (G/M) and after perfusion. **B.** Single-channel recordings of the mitoBK_Ca_ channel activity in symmetric 150/150 mM KCl isotonic solution (200 µM Ca^2+^) at +40 mV under control conditions, after the addition of 250 nM rotenone (Rot) and 5 mM G/M plus 250 nM Rot and after perfusion. **C.** Single-channel recordings of the mitoBK_Ca_ channel activity in symmetric 150/150 mM KCl isotonic solution (200 µM Ca^2+^) at +40 mV under control conditions, after the addition of 1 µM antimycin A (Anti) and 5 mM G/M plus 1 µM Anti and after perfusion. **D.** Analysis of the probability of channel opening at voltages ranging from −60 to +60 mV under the conditions described in A, B and C. *P<0.02 and **P<0.003 vs. the control.

Respiratory substrates such as L-glutamate plus L-malate (G/M), which lead to NADH synthesis, were used to modulate the mitoBK_Ca_ channel activity (Fig. 4). It was observed that 5 mM G/M decreased the P_o_ of the mitoBK_Ca_ channel at positive voltages. The effects were not reversible after perfusion with the control solution (n = 4) (Fig. 4A). The distribution of P_o_ at different voltages indicates that the changes were statistically significant at +20, +40 and +60 mV (Fig. 4D, left-hand panel). To identify the complexes of the respiratory chain that are involved, inhibitors of complex I (rotenone) and complex III (antimycin A) of the respiratory chain were used. Figure 4B and C show selected current-time traces of the mitoBK_Ca_ channel activity at +40 mV when the substrate (G/M) was added in the presence of rotenone and antimycin A (n = 3 for each experiment). The application of 250 nM Rot and 1 µM Anti alone did not affect mitoBK_Ca_ channel activity. Additionally, inhibitory effects of G/M substrates were not observed in the presence of rotenone or antimycin A. The analysis of the distribution of P_o_ is shown (Fig. 4D, middle and right panel).

### B3. Regulation of the mitoBK_Ca_ Channel by Succinate

**Figure 5:**
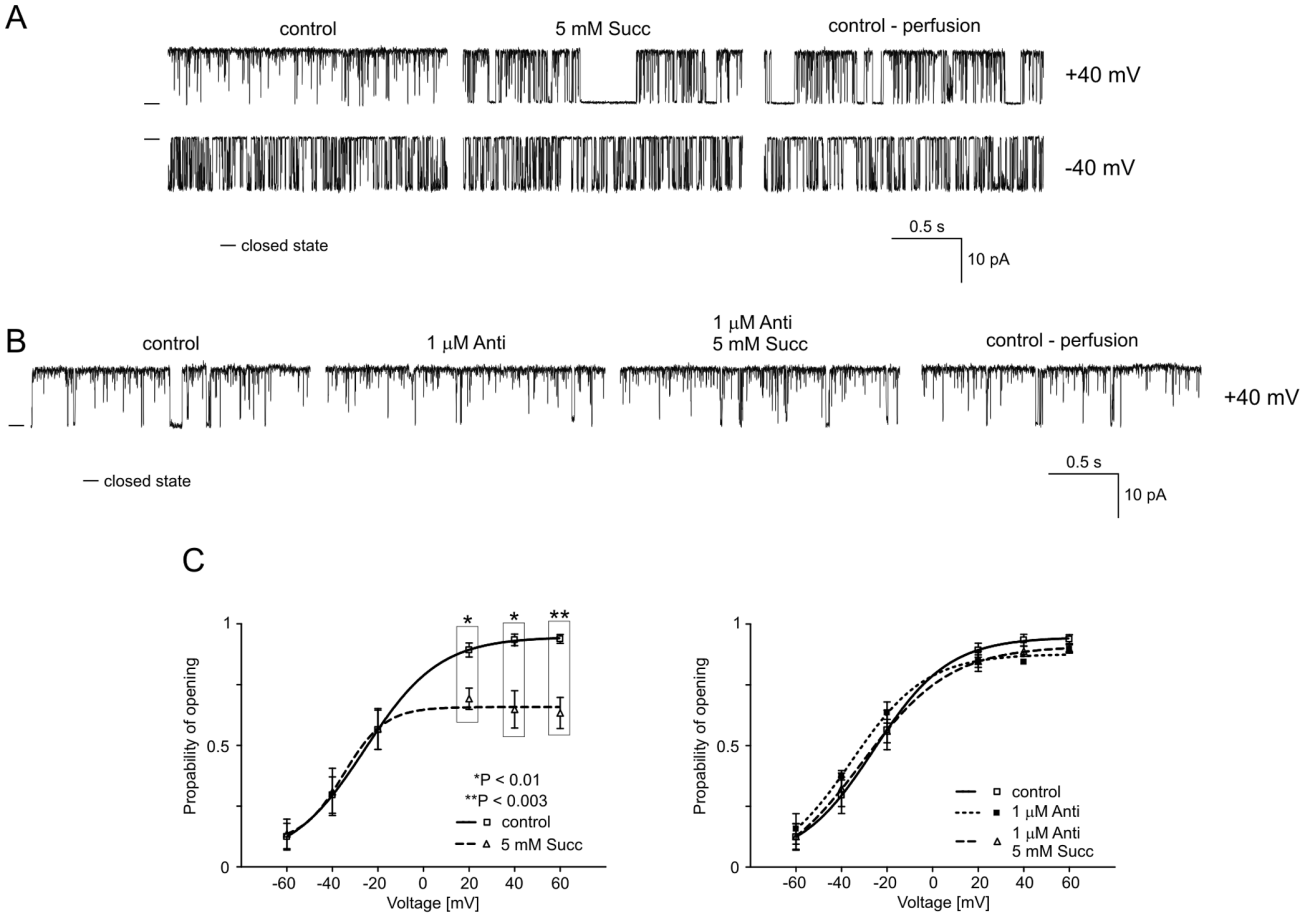
Succinate reduces the P_o_ of the mitochondrial large-conductance Ca^2+^-regulated potassium channel at positive voltages. **A.** Single-channel recordings of the mitoBK_Ca_ channel activity in symmetric 150/150 mM KCl isotonic solution (200 µM Ca^2+^) at +40 and −40 mV under control conditions, after the addition of 5 mM succinate (Succ), and after perfusion. **B.** Single-channel recordings of the mitoBK_Ca_ channel activity in symmetric 150/150 mM KCl isotonic solution (200 µM Ca^2+^) at +40 mV under control conditions, after the addition of 1 µM antimycin A (Anti) and 5 mM Succ plus 1 µM Anti, and after perfusion. **C.** Analysis of the probability of channel opening under the conditions described in A and B. *P<0.01 and **P<0.003 vs. the control.

To test for the possible involvement of complex II, succinate was used. Figure 5A shows typical single-channel records of the mitoBK_Ca_ channel activity in symmetrical isotonic solution at +40 and −40 mV after the addition of 5 mM succinate and after perfusion with the control solution. Similar to G/M in the previous experiments, succinate significantly decreased the P_o_ of the channel at positive voltages. These effects were reversible upon perfusion (n = 4) (Fig. 5C, left-hand panel). Both complex I and complex II could affect the mitoBK_Ca_ channel activity either directly or via the subsequent complexes. Therefore, the complex III inhibitor antimycin A (1 µM) was used before and during the application of succinate. Figure 5B presents selected current-time traces of the mitoBK_Ca_ channel activity at +40 mV (n = 3). Additionally, the dependence of P_o_ on the applied voltage is shown. Because the effects of succinate on the channel activity were not observed when antimycin A was present, it is likely that a later step modulates the mitoBK_Ca_ channel. Additionally, the distribution of the probability of channel opening is shown (Fig. 5C, right-hand panel).

### B4. Regulation of the mitoBK_Ca_ Channel by TMPD/ascorbate

**Figure 6:**
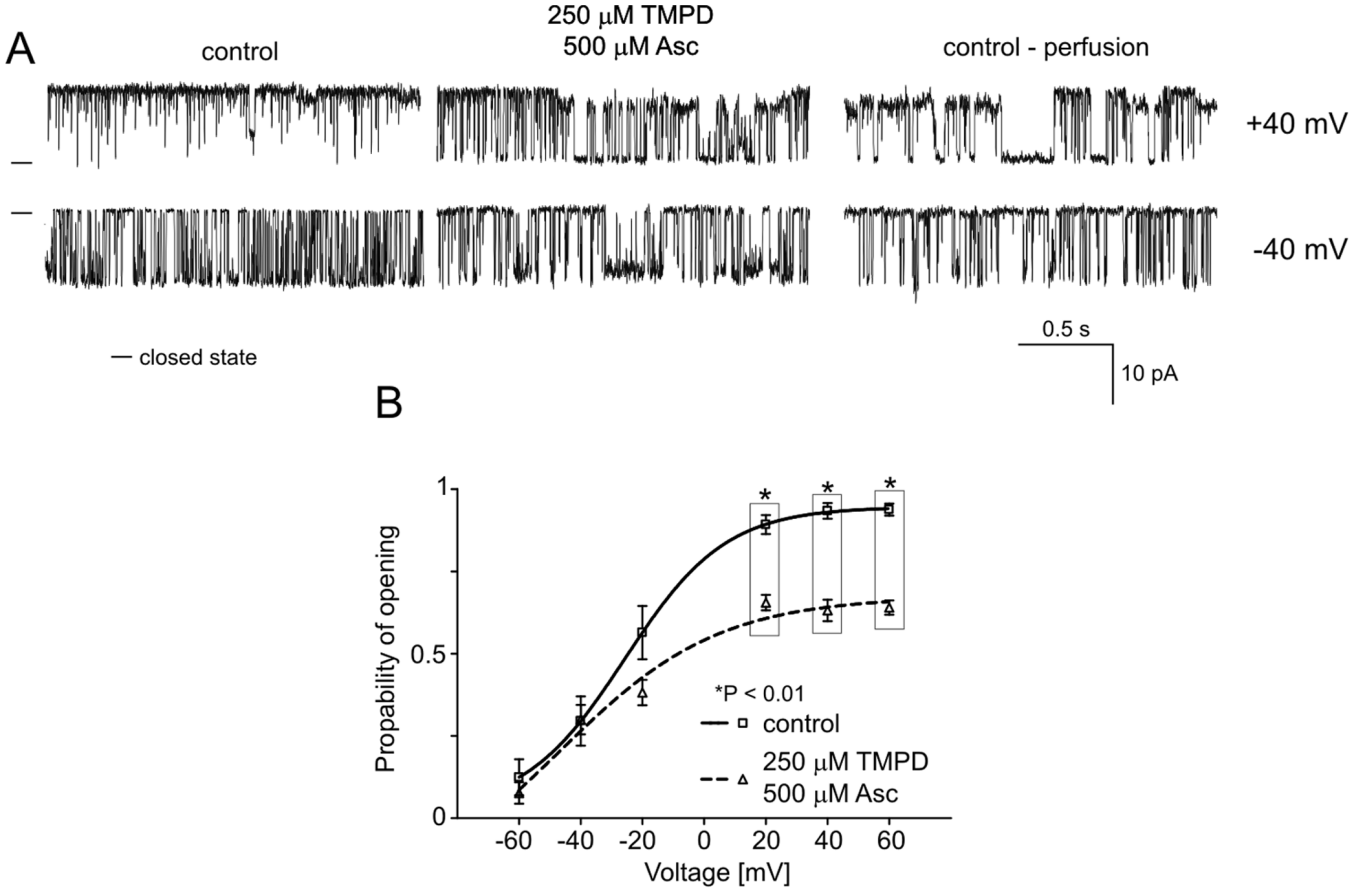
Effects of TMPD/ascorbate on mitoBK_Ca_ channel activity. **A.** Single-channel recordings of the mitoBK_Ca_ channel activity in symmetric 150/150 mM KCl isotonic solution (200 µM Ca^2+^) at +40 and −40 mV under control conditions, after the addition of 250 µM TMPD with 500 µM Ascorbate (Asc) and after perfusion. **B.** Analysis of the probability of channel opening under the conditions described in A. *P<0.01 vs. the control.

The next component of the respiratory chain is cytochrome c, which transfers electrons from complex III to cytochrome c oxidase (complex IV). To further investigate the changes in the mitoBK_Ca_ channel activity induced by the respiratory chain, the non-physiological reducing compound tetramethyl-p-phenylene diamine TMPD/ascorbate was used. As observed in the experiments with substrates of the respiratory chain enzymes, in the presence of TMPD/ascorbate (250 µM), the mitoBK_Ca_ channel activity was reduced (n = 4) (Fig. 6A). Additionally, the analysis of P_o_ revealed a decreased activity (∼40%) of the mitoBK_Ca_ channel at positive voltages (Fig. 6B).

### B5. Regulation of the mitoBK_Ca_ Channel by Potassium Cyanide

**Figure 7:**
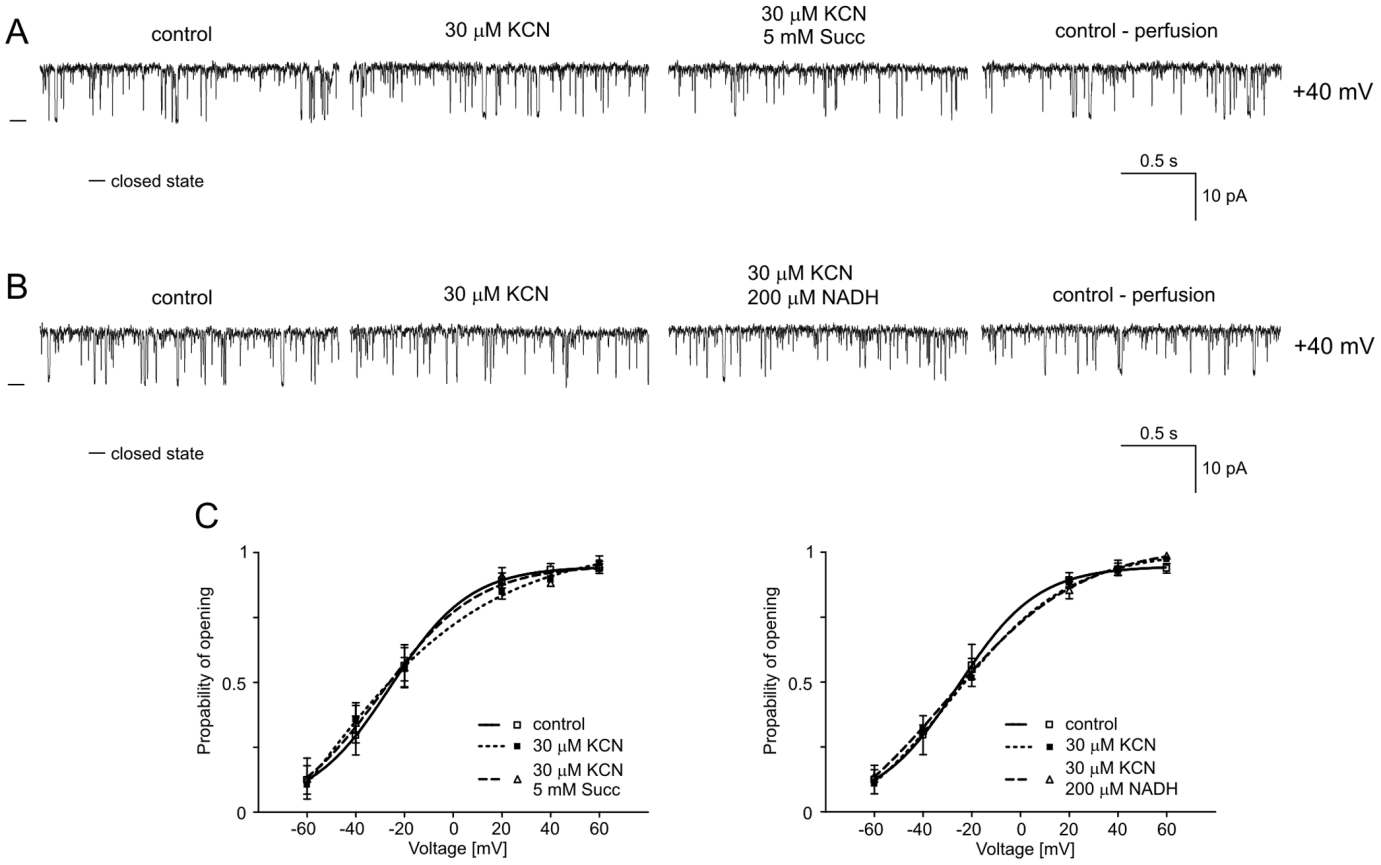
Cyanide abolishes the inhibitory effect of the respiratory chain substrates succinate and NADH. **A.** Single-channel recordings of the mitoBK_Ca_ channel activity in symmetric 150/150 mM KCl isotonic solution (200 µM Ca^2+^) at +40 mV under control conditions, after the addition of 30 µM KCN and 5 mM succinate plus 30 µM KCN, and after perfusion. **B.** Single-channel recordings of the mitoBK_Ca_ channel activity in symmetric 150/150 mM KCl isotonic solution (200 µM Ca^2+^) at +40 mV under control conditions, after the addition of 30 µM KCN and 200 µM NADH plus 30 µM KCN, and after perfusion. **C.** Distribution of P_o_ at different voltages under the conditions described in A and B.

Potassium cyanide (KCN) is a potent inhibitor of cellular respiration that acts on the mitochondrial cytochrome c oxidase (complex IV), blocking oxidative phosphorylation. After the effects of substrates of the respiratory chain on mitoBK_Ca_ channel activity had been established, KCN was used as an inhibitor of cytochrome c oxidase. Figure 7A and 7B show single-channel recordings of the mitoBK_Ca_ channel activity in symmetric isotonic solution at +40 mV after the addition of 30 µM KCN, 5 mM succinate plus 30 µM KCN (Fig. 7A) and 200 mM NADH plus 30 µM KCN (Fig. 7B) and after perfusion. The results of the analysis of P_o_ are presented in Fig. 7C. As observed for rotenone and antimycin A, cyanide prevented changes in the channel activity in the presence of substrates of the respiratory chain, such as succinate and NADH (n = 4). An analysis of the probability of channel opening was performed, and the results are presented in Figure 7C.

### C. Structural Coupling of the β4 Subunit to Cytochrome c Oxidase – Blue Native Electrophoresis

**Figure 8:**
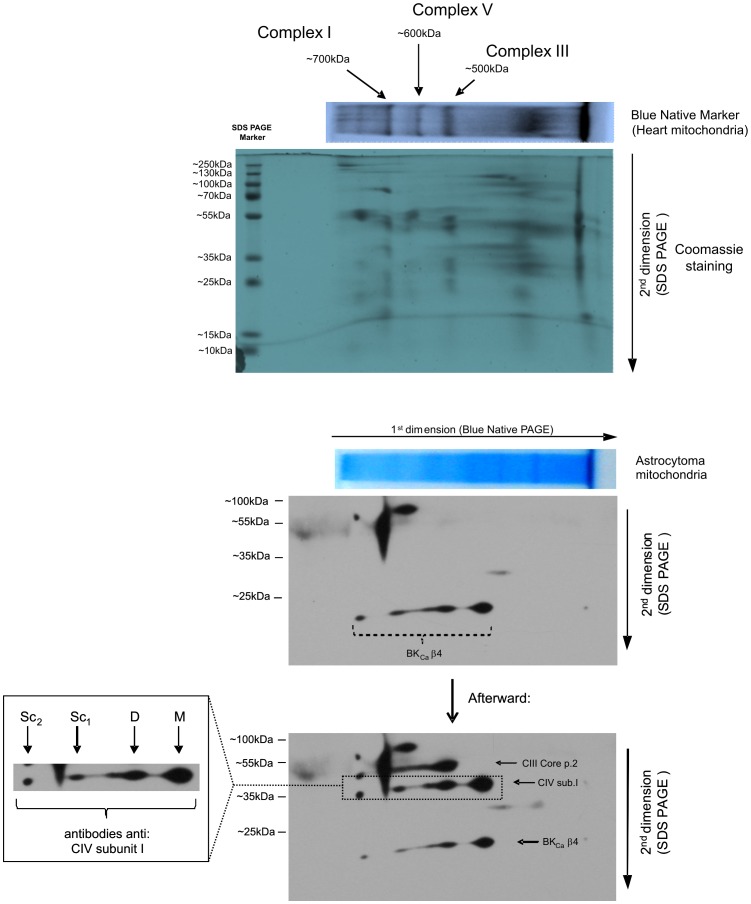
2D BN/SDS-PAGE separation of native astrocytoma mitochondria protein extracts. Two-dimensional separation was performed as described in the *Materials and Methods*, and the PVDF membrane was first immunoblotted for the BK_Ca_ channel β4 subunit (below, Coomassie staining panel). Next, the PVDF membrane was immunoblotted for the subunits of individual respiratory chain complexes (below the BK_Ca_ β4 panel). The BN-PAGE was calibrated based on the location of mitochondrial respiratory chain complexes that were isolated from rat heart mitochondria (above the panel for the blue native PAGE of mitochondria from astrocytoma cells). In the native astrocytoma lysate, mitochondria BK_Ca_ β4 co-localized with subunit I of cytochrome c oxidase. M, the monomeric form of cytochrome c oxidase; D, the dimeric form of cytochrome c oxidase; Sc_1_ and Sc_2_, complexes with higher molecular weights containing cytochrome c oxidase. A typical immunoblot from three separate experiments is shown.

Blue native electrophoresis (BNE) is a valuable tool for studying mitochondrial membrane protein complexes. To investigate whether the β4 subunit of the mitoBK_Ca_ channel interacts with cytochrome c oxidase, thus linking the mitochondrial respiratory chain and the mitoBK_Ca_ channel, we performed BNE, which was native for astrocytoma mitochondria, followed by SDS-PAGE. Spots on the immunoblot revealed that the BK_Ca_ channel β4 subunit localizes to the same lane as subunit I of cytochrome c oxidase (complex IV) (Fig. 8). This result indicates possible interactions between the β4 subunit and monomers, dimers and higher-molecular-weight complexes of mitochondrial complex IV of the respiratory chain.

## Discussion

Our results suggest putative functional and structural coupling of the respiratory chain via cytochrome c oxidase (complex IV) to mitochondrial large-conductance Ca^2+^-regulated potassium channels (mitoBK_Ca_ channel) in the human astrocytoma (glioblastoma) U-87 MG cell line.

The functional coupling of a voltage-generating complex (cytochrome c oxidase) with a voltage-dissipating complex (the mitoBK_Ca_ channel) could have a novel regulatory impact on the electro-chemical homeostasis of mitochondria. Hence, the fundamental questions that arise from our results not only concern the molecular mechanism but also the functional role of this type of coupling.

### Properties of the mitoBK_Ca_ Channel in Astrocytoma Cells

The mitoBK_Ca_ channel has been found in mitochondria from a human glioma cell line [1] and in mitochondria from cardiac [2], brain [4] and skeletal muscle [21]. Human astrocytoma cell lines have been previously used to study the properties of the mitoBK_Ca_ channel [18], [22], [23]. The mitoBK_Ca_ channel has pharmacological properties similar to those of the BK-type potassium channel previously identified in the plasma membranes of various cells. As for other mitochondrial potassium channels, it has been postulated that this channel plays an important role in the cytoprotection of various cells [13]. Recently, we identified a mitoBK_Ca_ channel in plant mitochondria [24].

Our study demonstrates that the β4 subunit is present in an astrocytoma cell line. It is likely that no other β subunits are present in this astrocytoma cell line. Previously, we identified various β subunits in neuronal tissue [5]. Additionally, we demonstrated the co-localization of β4-GFP with astrocytoma mitochondrial markers in the current study. Recently, it was postulated that the α subunit of the mitoBK_Ca_ channel is a DEC splice variant of the *Slo1* gene [25], [26].

### Putative Functional Coupling of the mitoBK_Ca_ Channel to Cytochrome c Oxidase

The putative functional coupling of the respiratory chain (generating a membrane potential ΔΨ) to electrogenic ion flow through membrane proteins, such as potassium channels, regulated by membrane potential appears to be obvious. The membrane potential regulates voltage-regulated potassium channels such as mitoBK_Ca_ channels and mitoKv channels [27]. Additionally, the mitochondrial potential modulates K^+^ flux via non-voltage-regulated pathways by changing the driving force for ion flux across the inner mitochondrial membrane. This system constitutes a type of global regulation via the macroscopic parameter ΔΨ.

In contrast to this type of global mechanism, our experiments suggest a direct structural-functional coupling of the respiratory chain to the mitochondrial potassium channel. These types of experiments were possible because the patch-clamp technique allows voltage-clamp in which ΔΨ is fixed independent of mitochondrial energization due to the presence of mitochondrial substrates. It would be difficult to perform such experiments with a suspension of isolated mitochondria and to assay the potassium flux in the presence or absence of mitochondrial substrates.

**Figure 9:**
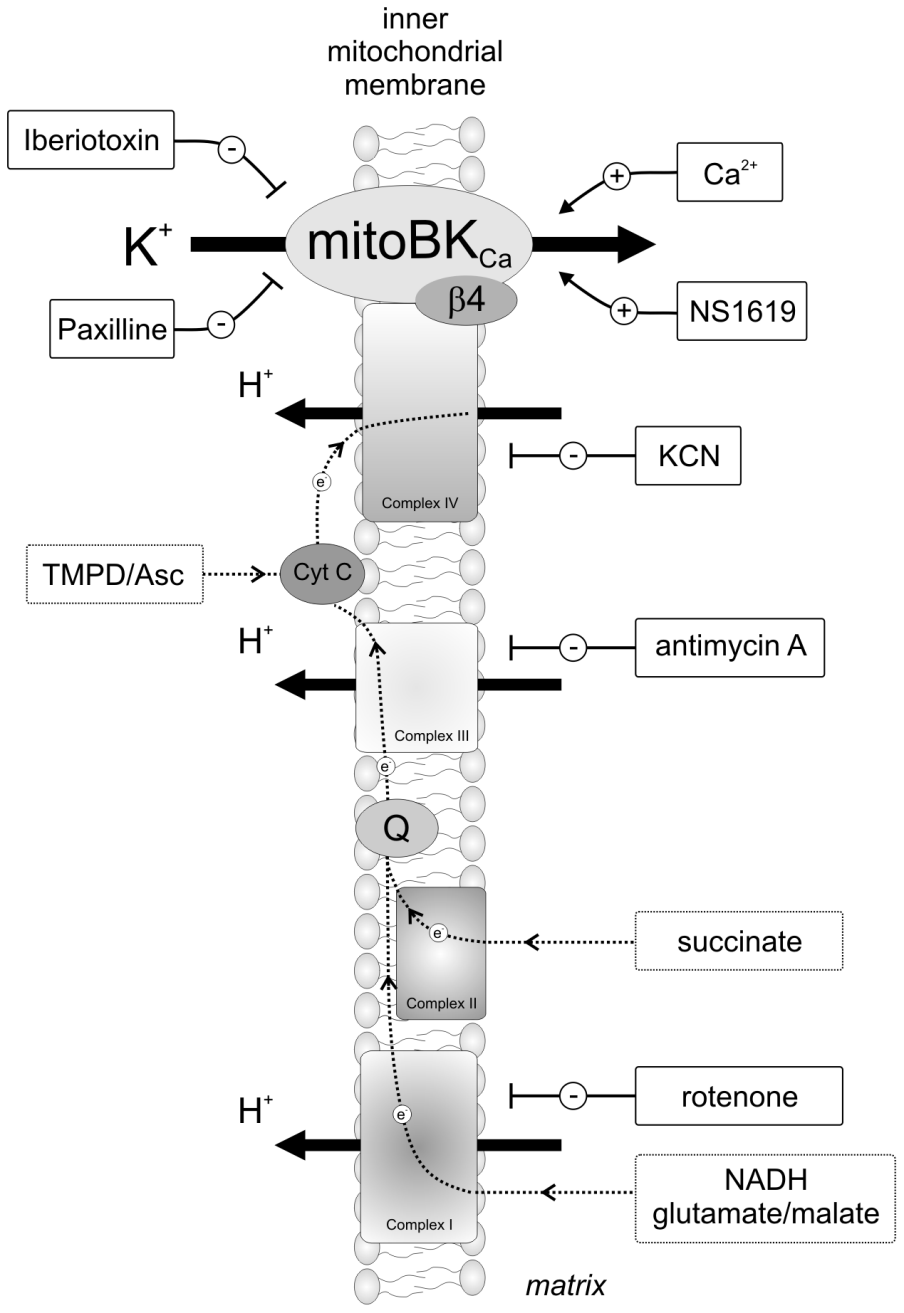
Schematic of the proposed model and the applied substrates/inhibitors of the respiratory chain and activators/blockers of the mitoBK_Ca_ channel. Complexes of the respiratory chain are shown, including NADH dehydrogenase (complex I), succinate dehydrogenase (complex II), ubiquinol cytochrome c oxidoreductase (complex III), and cytochrome c oxidase (complex IV). All of the substances (i.e., those that interact with the respiratory chain and with the mitoBK_Ca_ channel) that were used in this study are shown.

The results of experiments indicate that mitochondrial substrates inhibit the mitoBK_Ca_ channel by lowering its P_o_. This mechanism can be blocked by inhibitors of the respiratory chain, suggesting that a “redox signal” is transferred from the respiratory chain to the mitoBK_Ca_ channel via cytochrome c oxidase (complex IV). The proposed mechanism is shown in a simplified sketch (see Fig. 9). The details of molecular mechanism of this observation, however, remain unresolved. Probably, it is based on redox modulation of the mitoBK_Ca_ channel activity. Redox modulation of the plasma membrane BK channels was previously reported [28], [29], [30]. The redox regulation of the mitochondrial potassium channels such as mitoK_ATP_ in cardiocytes has also been observed recently [31]. Interestingly, the inhibitory effect remained when mitochondrial substrates were removed. Probably, a reduced respiratory chain is sufficient to inhibit the mitoBK_Ca_ channel but channel activation could be obtained by external oxidative stress. Activation of the mitoK_ATP_ channel by superoxide was recently shown [31], [32].

### Putative Structural Interaction of Cytochrome c Oxidase and the mitoBK_Ca_ Channel

Experiments using mitochondrial substrates (NADH, succinate, malate/glutamate), artificial electron donors (TMPD/ascorbate) and specific inhibitors of mitochondrial complexes (rotenone, antimycin A, KCN) not only suggested that mitoBK_Ca_ channels are regulated by the respiratory chain but also indicated that cytochrome c oxidase is involved in the "signal transduction" from the respiratory chain to the mitoBK_Ca_ channel. The results of the BNE experiments support these conclusions as the β4 subunit of mitoBK_Ca_ co-migrated with cytochrome c oxidase. The BNE technique has been used previously to identify interactions between various proteins.

It has been suggested that super-complexes of mitochondrial respiratory chain enzymes can form a mitochondrial ion channel [33]. Based on the observations described in this paper, one could also speculate that the isolated complexes were mixed with potassium channels due to the specific interaction of the mitochondrial potassium channels with respiratory chain enzymes.

Furthermore, it was recently shown via a yeast two-hybrid assay that the β1 subunit of the BK channel from cardiac myocytes interacts with cytochrome c oxidase subunit I. The results of past immunocytochemical experiments have also demonstrated that the β1 subunit interacts with cytochrome c oxidase and co-localizes with rat cardiac mitochondria [34].

### The Role of the Interaction between the Respiratory Chain and Potassium Channels

Why would the respiratory chain lower the P_o_ of the mitoBK_Ca_ channel in the presence of substrates, and what is the physiological significance of such a response?

There are a few possible explanations. First, preventing the K^+^ influx during the energization of mitochondria (in the presence of substrates) by decreasing the activity of the mitoBK_Ca_ channel would cause hyperpolarization of the mitochondria ΔΨ. Thus, reducing the K^+^ flux would allow ΔΨ reaching a specific value faster. Second, the strong driving force of a large ΔΨ would favor the influx of K^+^ ions by itself. A strong influx would disrupt the potassium balance, which is vital for controlling the mitochondrial volume. Therefore, a decrease of activity mitoBK_Ca_ channel would protect mitochondria from an overload with potassium ions and would thus prevent the disruption of mitochondrial volume homeostasis. In other words, a lack of coupling between complex IV and the mitoBK_Ca_ channel would not allow the inhibition of the K^+^ channel activity upon mitochondrial energization.

Recently, it was shown that hypoxia increases the activity of the BK_Ca_ channel in the inner mitochondrial membrane and reduces the activity of the permeability transition pore (PTP) [18]. This may suggest a functional coupling between mitoBK_Ca_ channel and PTP. Potassium channel activation may lead to a lower Ca^2+^ uptake through the Ca^2+^ uniporter. Hence, channel activity lowered by mitochondrial substrates (as described in this report) may support PTP activation leading to cell death. It could be interesting too, to explore a possible coupling of the respiratory chain to the Ca^2+^ uniport, now known to be a Ca^2+^ channel [35].

### Final Remarks

In summary, our results support the existence of a structural and functional interaction between the mitoBK_Ca_ channel and cytochrome c oxidase. Together with the modulation of the channel by Ca^2+^ and ΔΨ, these observations reveal a previously unknown pathway for the regulation of mitoBK_Ca_ channels.

Our observations raise the following open questions:

What is the mechanism of coupling? Is the mechanism based on a structural or a chemical redox signal that modulated the mitochondrial potassium channel based on the redox status of the respiratory chain?
Is the channel inhibition mediated by the respiratory chain reversible?
Which subunits of cytochrome c oxidase are involved in this coupling?
Does coupling occur only via the β subunits of the K-channel or does are the α subunits involved?
How do the substrates of the respiratory chain interfere with the Ca^2+^ regulation of the channel?
Are other mitochondrial potassium channels, such as the ATP-regulated and voltage-dependent potassium channels, regulated by similar modes of channel modulation?
To what extent are the observed properties unique for astrocytoma mitochondria?
Is there a similar type of coupling for mitochondrial anion channels?

All of these questions should be addressed in future studies.
